# Alström syndrome: a paradigm for diffuse fibrosis and clinical progression

**DOI:** 10.1186/1532-429X-15-S1-P159

**Published:** 2013-01-30

**Authors:** Nicola C Edwards, William E Moody, Emma Springthorpe, Peter Weale, Richard Paisey, Tarekegn Geberhiwot, Richard Steeds

**Affiliations:** 1 Cardiovascular Medicine, University of Birmingham & Queen Elizabeth Hospital Birmingham, Birmingham, UK; 2 Cardiovascular Medicine, University of Birmingham & Queen Elizabeth Hospital Birmingham, Birmingham, UK; 3 Cardiology, Queen Elizabeth Hospital Birmingham, Birmingham, UK; 4 Applications Specialist, Siemens Healthcare UK, Camberley, UK; 5 Endocrinology, Torbay Hospital, Torquay, UK; 6 Endocrinology, University of Birmingham & Queen Elizabeth Hospital Birmingham, Birmingham, UK; 7 Cardiology, University of Birmingham & Queen Elizabeth Hospital Birmingham, Birmingham, UK

## Background

Alström syndrome (ALMS) is a rare autosomal recessive genetic disorder characterised by progressive endocrine disarray, sensorineural deficit, cardiac, renal, and hepatic abnormalities. Idiopathic infantile dilated cardiomyopathy (CMP) is common, presenting acutely in 45% of individuals and recurs or develops de novo in 65% of adolescents with high rates of morbidity and mortality. Myocardial fibrosis has been demonstrated at post-mortem and on MRI with patchy diffuse late gadolinium enhancement (LE) in an older cohort of ALMS patients. We hypothesise that subclinical diffuse fibrosis in young patients with ALMS precedes any change in conventional parameters of ventricular function or overt scarring on LE.

## Methods

**Figure 1:**
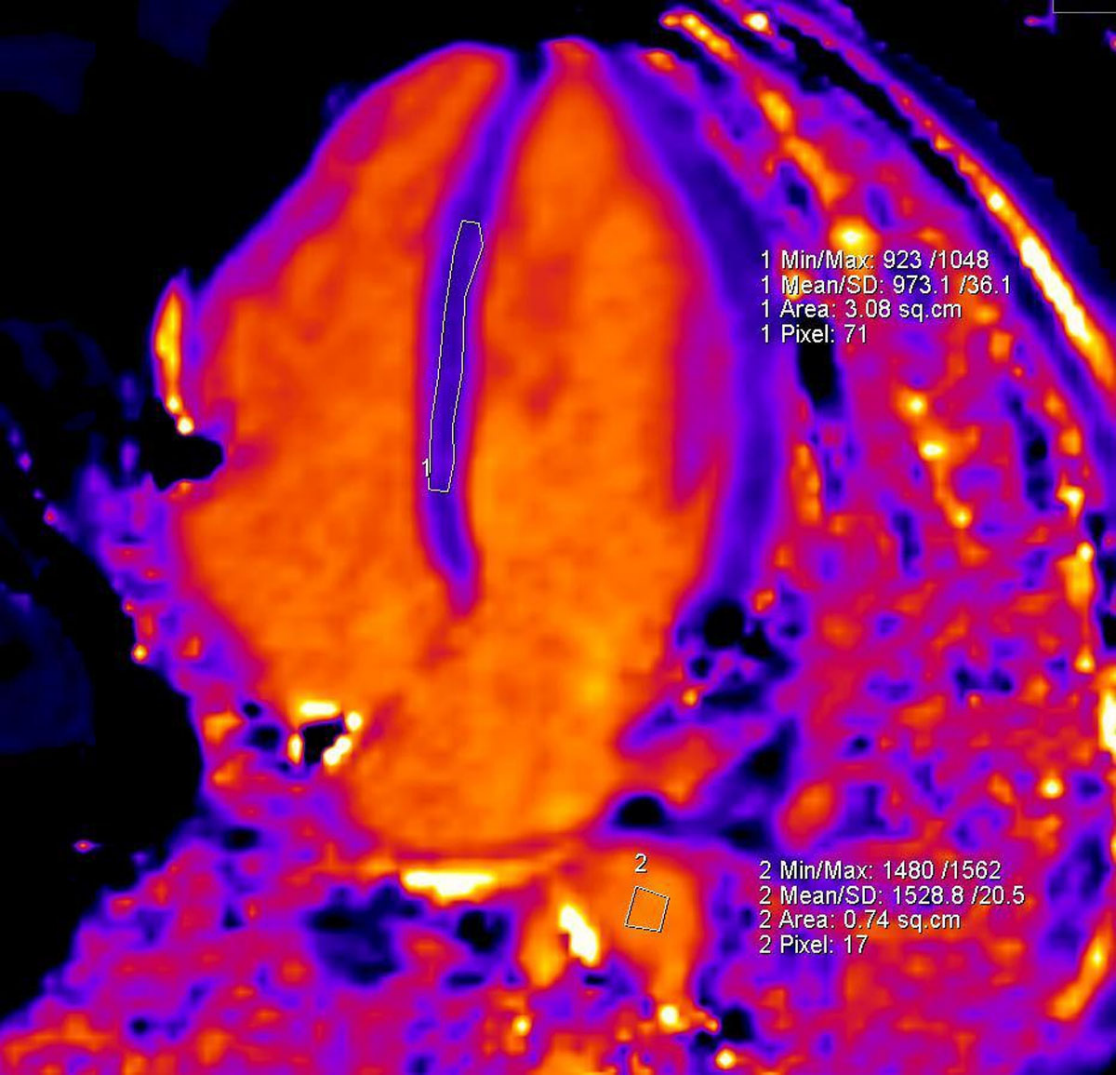
Pre-contrast MOLLI with the regions of interest drawn in the basal and mid septum and descending aorta

**Figure 2:**
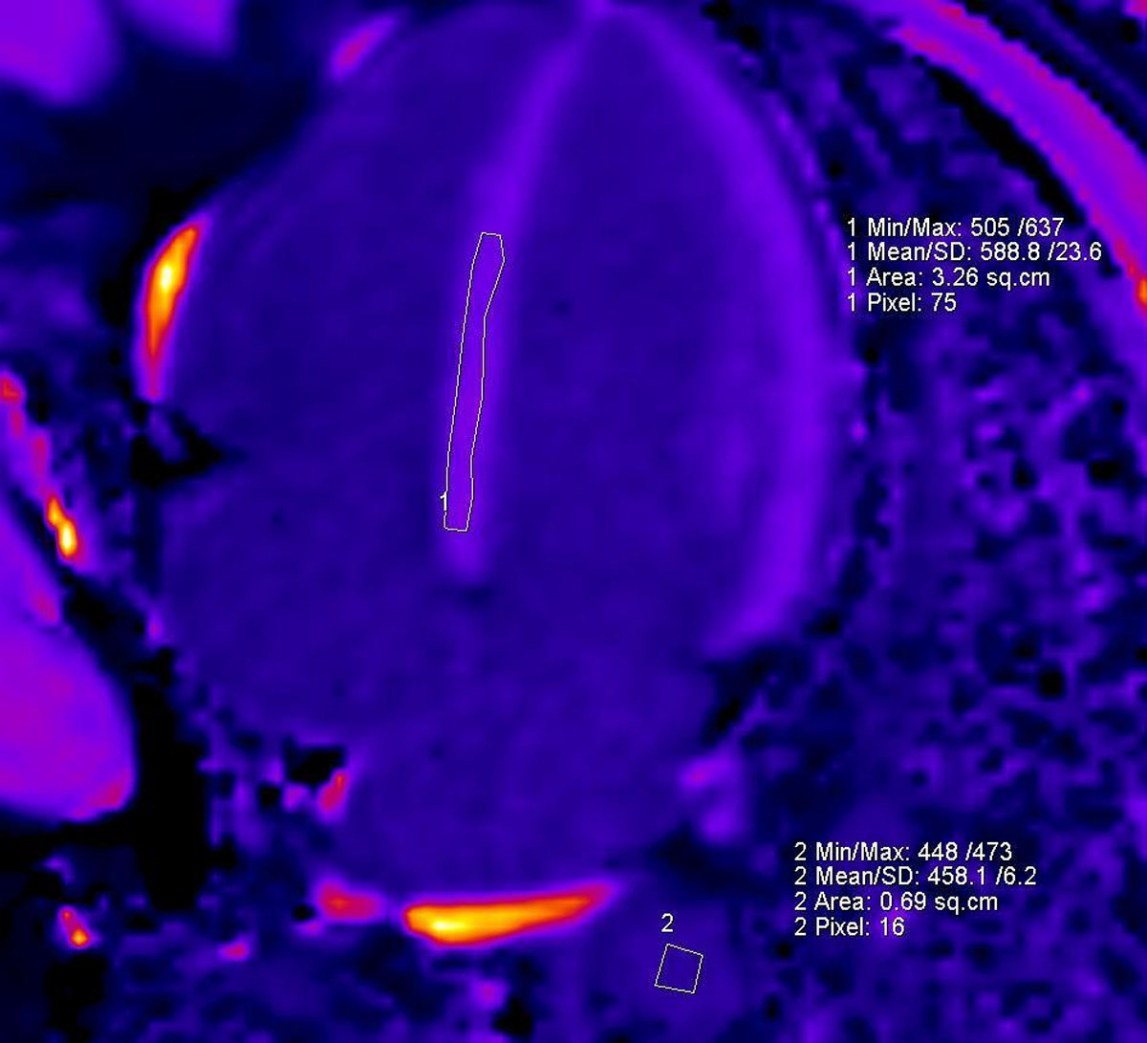
MOLLI 15 minutes after contrast with the regions of interest drawn in the basal and mid septum and descending aorta

Thirteen patients (mean age 27 +/- 11 years, 77% male, prevalence infantile CM 15%) from the National Specialist Commissioning Group for Alström Syndrome, Birmingham, UK and matched age and gender controls were prospectively assessed with 2D echocardiography and cardiac MRI (1.5T). Late gadolinium enhancement images were acquired 5-7 minutes after contrast. Myocardial extracellular volume (ECV) was assessed using T1-mapping pre and 15 minutes post gadolinium (0.1mmol/Kg) using a modified look-locker inversion recovery sequence (MOLLI) (Figures 1 &2). Myocardial T1 was measured in the basal and mid septum avoiding areas of LE.

## Results

Septal myocardial ECV was increased in female ALMS compared to male ALMS (0.27 +/- 0.02 vs. 0.31 +/- 0.02, p<0.05). Septal myocardial ECV was increased in ALMS compared to controls (0.26 +/- 0.02 vs. 0.29 +/- 0.03, p<0.05). Three male older ALMS patients (mean 43 +/- 5 years vs. 27 +/- 10 years) all without a history of infantile CMP, had patchy diffuse LE in non-coronary artery territories with an increased ECV compared to remote "normal" myocardium (ECV 0.41 +/- 0.08 vs. 0.27 +/- 0.03, p<0.05). There were no differences in mean LV mass, LV ejection fraction or LA volume between ALMS and controls. Increased septal myocardial ECV correlated with a reduction in lateral and septal TDI systolic velocities (r = -0.82, p<0.05, r = -0.81, p<0.05). NT-BNP was not correlated with septal ECV but was increased in patients with LGE (median 178 pmol/L vs. 44 pmol/L).

## Conclusions

Patients with ALMS demonstrate a spectrum of myocardial fibrosis, which correlates with reduced longitudinal contractility before changes in conventional markers of LV structure and function. This precedes a clinically significant increase in BNP that appears with overt LE and suggests that increased ECV may be a target for early modification of pharmacological therapy.

## Funding

National Commissioning Group

